# New Insights into The Photoactivity of Shape-Tailored BiVO_4_ Semiconductors via Photocatalytic Degradation Reactions and Classical Reduction Processes

**DOI:** 10.3390/molecules25204842

**Published:** 2020-10-20

**Authors:** Zsolt Kása, Enikő Eszter Almási, Klára Hernádi, Tamás Gyulavári, Lucian Baia, Gábor Veréb, Zsuzsanna László, Zsolt Pap

**Affiliations:** 1 Institute of Environmental Science and Technology, University of Szeged, Tisza Lajos blvd. 103, H-6725 Szeged, Hungary; kasa.zsolt@chem.u-szeged.hu (Z.K.); almasieniko@geo.u-szeged.hu (E.E.A.); 2 Vulcano Research Group, Department of Mineralogy, Geochemistry and Petrology, University of Szeged, Egyetem Street 2, H-6722 Szeged, Hungary; 3 Department of Applied and Environmental Chemistry, University of Szeged, Rerrich Béla sqr. 1, H-6720 Szeged, Hungary; hernadi@chem.u-szeged.hu (K.H.); gyulavarit@chem.u-szeged.hu (T.G.); 4 Nanostructured Materials and Bio-Nano-Interfaces Center, Interdisciplinary Research Institute on Bio-Nano-Sciences, Babeș-Bolyai University, Treboniu Laurian str. 42, RO-400271 Cluj-Napoca, Romania; 5 Faculty of Physics, Babeș-Bolyai University, M. Kogălniceanu str. 1, RO-400084 Cluj-Napoca, Romania; 6 Faculty of Engineering, Institute of Process Engineering, University of Szeged, Moszkvai Blvd. 9, H-6725 Szeged, Hungary; verebg@mk.u-szeged.hu (G.V.); zsizsu@mk.u-szeged.hu (Z.L.)

**Keywords:** bismuth vanadate, shape-tailoring, oxalic acid, rhodamine B, photodegradation

## Abstract

In the present study, additive-free, pH-driven, hydrothermal crystallization was used to obtain shape-tailored monoclinic BiVO_4_ photocatalysts. The as-prepared BiVO_4_ products were systematically characterized, uncovering their crystallographic, morphologic and optical properties, while their applicability was verified in the visible light-driven photodegradation of oxalic acid and rhodamine B. Monoclinic clinobisvanite was obtained in most cases, with their band gap values located between 2.1 and 2.4 eV. The morphology varied from large, aggregated crystals, individual microcrystals to hierarchical microstructures. It was found that the degradation efficiency values obtained in the case of oxalic acid were directly related to the presence of (040) crystallographic plane, while the degradation of rhodamine B was partially independent by the presence of this structural feature. The importance of (040) crystallographic plane was also demonstrated via the reduction of Cu^2+^ to Cu, by analyzing the Raman spectra of the Cu containing samples, the mean primary crystallite size of Cu and Cu content. Furthermore, the presence of (040) crystallographic plane was directly proportional with the hydrodynamic properties of the powders as well.

## 1. Introduction

Advanced oxidation processes (AOPs) can offer viable solutions to remove persistent organic pollutants such as dyes from water and wastewater [1,2], which have been emitted mostly by textile industries [3]: methylene blue, methyl orange, congo red, malachite green and rhodamine B are the most frequent ones found [4]. These compounds are removable by heterogeneous photocatalysis, which is based on semiconductor materials, and a specific light source: UV, visible or sunlight [5]. Nowadays, photocatalysis focuses on solar energy exploitation and conversion including degradation of organic pollutants [6], residual pharmaceuticals/drugs [7], dyes [8,9,10,11], inactivation of bacteria [12], water splitting [13], etc.

Titanium dioxide is by far the most investigated photocatalyst for different applications [14,15,16,17], but it is limited by the relatively wide band gap (e.g., rutile = 3.2 eV), therefore many attempts were made to extend its action spectrum to the visible light region [18] such as doping with other elements [19], sensitization with dye molecules [20] or composites with noble metals [21] or other materials [22]. A different approach could be to use another semiconductor for water purification, which can be excited under visible light, such as bismuth tungstate or bismuth vanadate [23,24].

Bismuth vanadate (BiVO_4_) has attracted a great deal of attention as a novel photocatalyst with the potential of excellent photocatalytic performance under visible light irradiation. Bismuth vanadate has three crystal phases: tetragonal zircon, tetragonal and monoclinic scheelite structure [25]. Among these, the monoclinic crystal phase has the best photocatalytic activity under visible light irradiation [26]. Several preparation methods have been reported for the fabrication of monoclinic BiVO_4_ microstructure, e.g., sonochemical [27] or electrochemical reactions [28], hydrothermal crystallization [29], sol-gel method [30] or ultrasonic spray pyrolysis [31].

It is known that the crystal shape [25], particle size [32], specific surface area or simply the crystallographic plane ratio [33] is crucial for improving the photocatalytic activity of BiVO_4_. These properties are closely correlated to each other, and these parameters can be fine-tuned with different solvents and additives such as ethylene glycol [34] and sodium dodecyl benzene sulfonate [35], or simply by pH modulation [36,37].

Moreover, bismuth vanadate can be applied in the production of molecular oxygen, and in water splitting as well [38]. In the case of bismuth vanadate, the (040) crystal facet has enhanced reactivity, compared to other ones, because the photogenerated holes tend to migrate towards the (040) crystal facet and generate reactive radicals, or oxidize the adsorbed organic pollutants by direct hole oxidation [27,39].

Nevertheless, as shown above, diverse scientific data are available concerning the photocatalytic processes carried out in the presence of shape-tailored BiVO_4_. Moreover, specific shape manipulation approaches were also used successfully, such as the already mentioned pH manipulation, to obtain different crystal shapes. Despite the relatively vast literature available on this subject, evidence is needed on the crystal shapes’ effect on different model pollutants’ degradation (which show different degradation routes).

Hence, in this study, monoclinic BiVO_4_ catalysts were prepared by hydrothermal crystallization, and the effect of the pH of the crystallization medium was investigated in connection with the microcrystals’ shape, crystal phase, band gap and photocatalytic activity. In addition, the role of different crystallographic planes was investigated, using both photocatalytic and classical reaction approaches (elemental Cu deposition). Most importantly, a comparison was made between the degradation of two different model pollutants, which are known to have different degradation pathways (oxalic acid and rhodamine B). More importantly it should be mentioned that this type of study, which investigates the importance of the synthesis conditions in the frame of catalytic and non-catalytic approaches, is still rare and more are needed to avoid synthesis related issues and to obtain applicable materials in different research fields.

## 2. Results and Discussion

### 2.1. Crystal Phase Composition and Crystal Size

**Figure 1:**
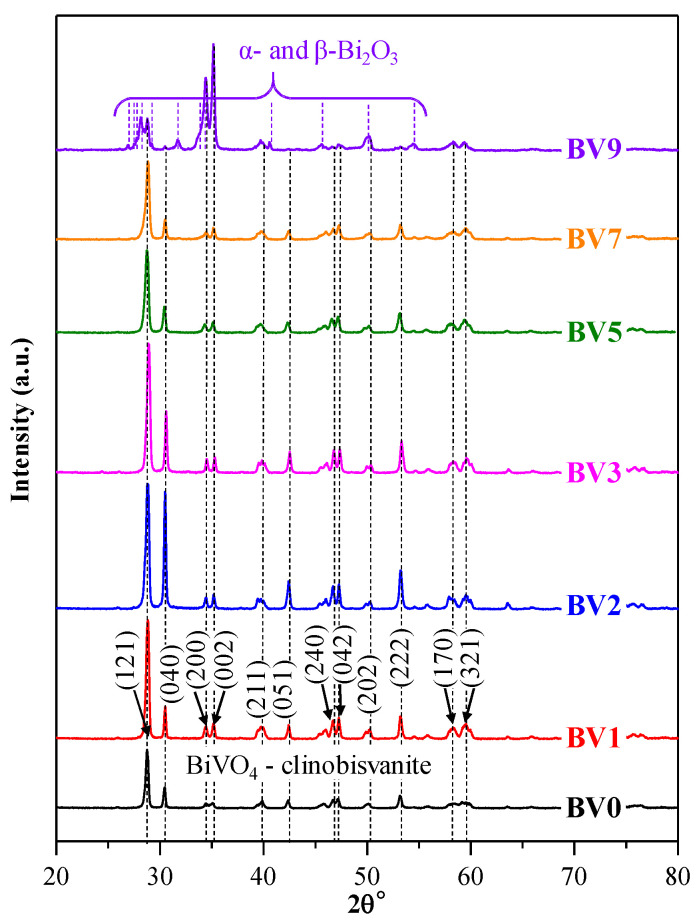
The XRD patterns of the as-obtained photocatalysts, showing the presence of the monoclinic crystal phase, the only exception being sample BV9, where α- and β-Bi_2_O_3_ were also detected.

XRD was used to analyze the crystal structure of the BiVO_4_ samples (Figure 1). The diffraction peaks of BiVO_4_ (except in the case of sample BV9) were identified as monoclinic BiVO_4_: 28.84°, 30.49°, 34.44°, 35.14°, 39.78°, 42.40°, 46.77°, 47.08°, 50.13°, 53.17°, 58.31° and 59.35° (JCPDS No. 14-0688). These signals corresponded to (121), (040), (200), (002), (211), (051), (240), (042), (202), (222), (170) and (321) crystallographic planes, respectively, as shown in Figure 1.

All diffraction peaks were sharp and intense, demonstrating the highly crystalline character of the samples. It can be noted that the ratio of the first (121) and second (040) diffraction peaks changed with the pH increase. Initially, the intensity of the (040) diffraction peak increased in intensity until pH = 2, after which the trend was reversed. When the pH was set to 9 (BV9), the (040) crystallographic plane’s signal completely disappeared, while the other main diffraction peak (121) was greatly reduced. Simultaneously, the diffraction peak intensities of (200) and (002) were increased, however showing the sign of multiple peaks overlapping there. Moreover, minor signals appeared at 26.94°, 28.14°, 31.66° and 41.58°, which can be attributed to a mixture of α- and β-Bi_2_O_3_ (COD No. 00-101-0004 and 00-901-2328). Both oxides can be obtained using basic crystallization conditions as it was our case [40], therefore a further increase of the pH was not considered.

All samples’ average primary crystallite size values were calculated using the Scherrer equation, and it was found that, without the addition of NaOH, the individual crystallite mean size was 42.8 nm (BV0), but after addition of NaOH in the synthesis, a decrease was observed. This trend was valid until pH = 5 (in sequence: 37.4, 36.3, 36.9 and 29.8 nm, from BV1 to BV5, respectively). After that, the values started to increase again (33 nm for BV7 and 43.4 nm for BV9). These results indicate that the pH modulation could be appropriate for the formation of a single-phase monoclinic crystal structure, controlling the crystal facet ratio and the size of the individual crystals. However, caution should be taken, as the SEM micrographs may show micrometric crystals, therefore the calculated crystallite mean size values may be inaccurate.

### 2.2. Morphological Characterization

**Figure 2:**
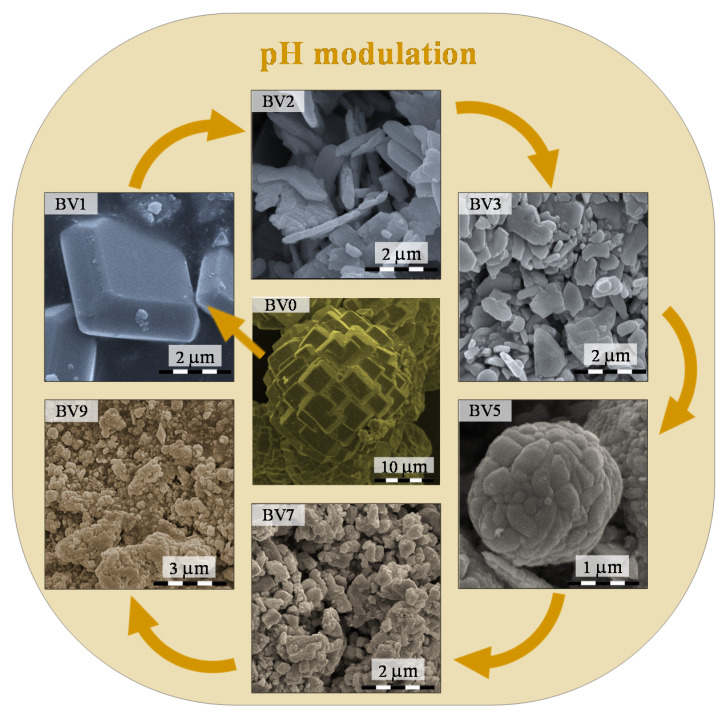
SEM micrographs of the investigated samples, showing the changes in the morphology as the pH of synthesis mixture was adjusted.

All bismuth vanadate samples were characterized in terms of morphology (Figure 2) to find out more about the influence of the pH. The SEM micrographs of BV0 showed that this sample was made of large crystals (d > 5–10 µm), built from individual thick plates (d ~ 3–5 µm).

When the pH value reached 1 (BV1), the individual plate size did not change (d ~ 3 µm), but the aggregation degree of the crystals was significantly lower, and more and more individual plates appeared. At pH 2 (BV2) and pH 3 (BV3), single sheets started to form (Figure 2). In the case of BV3, a thickening process started. Furthermore, the crystals were between 50 and 1000 nm. In the case of BV5, the above-mentioned trend did not continue; the polycrystallinity of the sample increased, while a secondary, “cauliflower-like” shape was formed (d ~ 2 µm). For BV7 and BV9, this secondary structure transformed, while no specific morphology was observed.

### 2.3. Optical Properties

**Figure 3:**
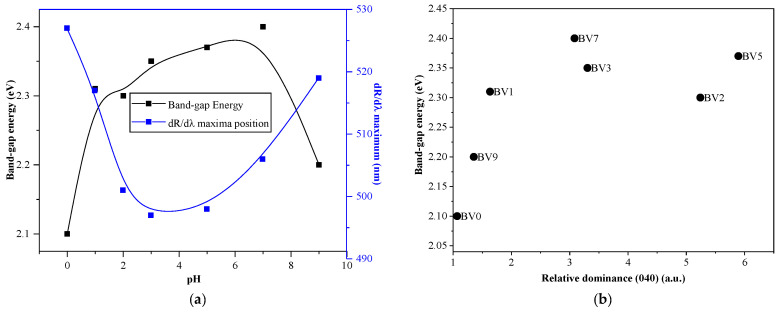
The band-gap value and the first derivative DRS spectra maxima changes in function of the synthesis pH (**a**). The band-gap value changes as a function of (040) crystallographic plane’s dominance (**b**).

All samples’ optical properties were investigated by UV-Vis reflectance spectroscopy. From the DRS spectra, the band-gap values were calculated using the Kubelka-Munk equation (Table 1). The maximum band-gap value was reached at pH = 7 (2.4 eV for BV7, Figure 3). However, a more detailed interpretation could be carried out using the first derivative reflectance spectra (dR/dλ), which could indicate the real band gap values and possible electron transition wavelengths. The changes observed in the maximum of the dR/dλ were also plotted, against the synthesis pH, and it was found that it followed the same trend as the band gap values (as the dR/dλ values were given in nanometers, the same trend means here an asymmetric curve), pointing out that the electron transitions will take place at the determined band gap energies (this was necessary as the band gap values are not always comparable with the photocatalysts’ real excitation wavelength [41]). As the pH adjustment already showed a significant impact on the crystal orientation and the optical properties, a correlation between the orientation of the crystal and the band-gap was expected.

It was found that, with the increase of (040) crystallographic plane’s dominance, as it was shown in the XRD patterns of the samples, the band-gap values increased abruptly from 2.1 eV, achieving finally 2.4 eV. It seems that trough the modification of the crystal orientation, the band-gap can be tuned as well, which is also known from (001) facet dominated anatase titania photocatalysts [42]. This approach also could be the key for determining the photoactivity of the materials.

### 2.4. Photocatalytic Activity under Visible Light Irradiation

**Figure 4:**
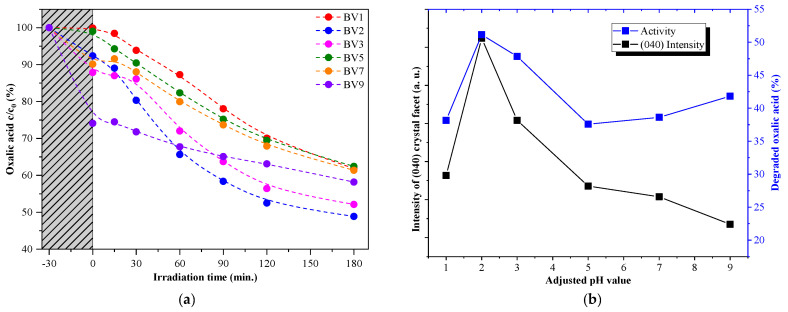
The photocatalytic activity of the samples in the degradation of oxalic acid under visible light irradiation (**a**). The activity trend similarity with the (040) crystallographic plane’s dominance trend (**b**). Please note that the lines used to connect the data points are only there to increase the readability of the figure and do not represent any mathematical trends or equation fittings.

**Figure 5:**
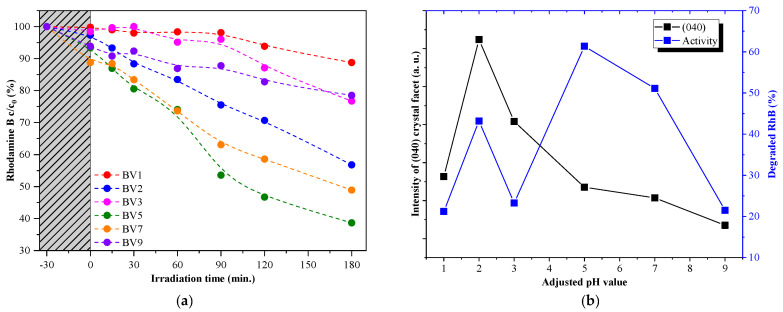
The photocatalytic activity of the samples in case of rhodamine B under visible light irradiation (**a**) and the activity trend similarity with the trend of (040) crystallographic plane’s dominance (**b**). Please note that the lines used to connect the data points are only there to increase the readability of the figure and do not represent any mathematical trends or equation fittings.

All the samples’ photocatalytic activity was investigated to correlate the pH tuning effects on the photocatalytic degradation of oxalic acid and rhodamine B (Figure 4 and Figure 5). All samples were active in the degradation of oxalic acid (Figure 4). Sample BV1 exhibited the lowest adsorption capacity, while the sample BV9 showed the highest one (0.9% and 33.7% of oxalic acid adsorbed). Interestingly, BV2 showed the highest photoactivity (51.1%). When the photoactivity values were correlated with the crystal orientation and the pH of the synthesis, an interesting trend was detected. The calculated (040) crystallographic plane’s relative diffraction peak intensity varied in the same way as the observed photoactivity (Figure 4). This shows that the degradation process is strongly dependent on the presence of (040) crystallographic plane (Figure 4).

By looking at the results achieved from the rhodamine B degradation experiments, one can see that the adsorption on the surface of the catalysts was lower than that exhibited in the case of oxalic acid. However, the obtained activity trend was different. For samples BV1 and BV2, an activity increase was observed (from 21.2% to 43.2%), which was similar to the trend observed in the case of oxalic acid, but at higher pH values (samples BV5 and BV7): the degradation capacity does not follow the (040) diffraction peak’s relative intensity, showing an opposite trend (there is one exception to this behavior, sample BV3).

These results were partially expected, as the degradation pathway of these two model compounds differs:

- Oxalic acid is usually degraded by a direct hole oxidation mechanism, as this compound can be easily adsorbed on the surface of several photocatalysts [43].
- The degradation of rhodamine B starts with an electron transfer step between the dye molecule and the photocatalyst. In addition, direct hole oxidation is an option as stated in the literature [44].

Considering the arguments presented above, the following presumption can be made. As (040) is considered to be the crystallographic plane responsible for the oxidation process, it is highly plausible that those materials which show a specific growth of this facet will oxidize oxalic acid more efficiently. This was not valid for rhodamine B.

As we have a small contradiction in the photocatalytic results, another confirmation was needed, using other reaction types, instead of photocatalytic degradation. The most convenient was to use a reaction which can shed some light on the mentioned aspect. Therefore, the reduction process of Cu^2+^ was chosen in the presence of BiVO_4_. It is a known reaction which does not occur without the presence of a catalyst.

### 2.5. Deposition of Cu on the Surface of the Investigated Photocatalysts

**Figure 6:**
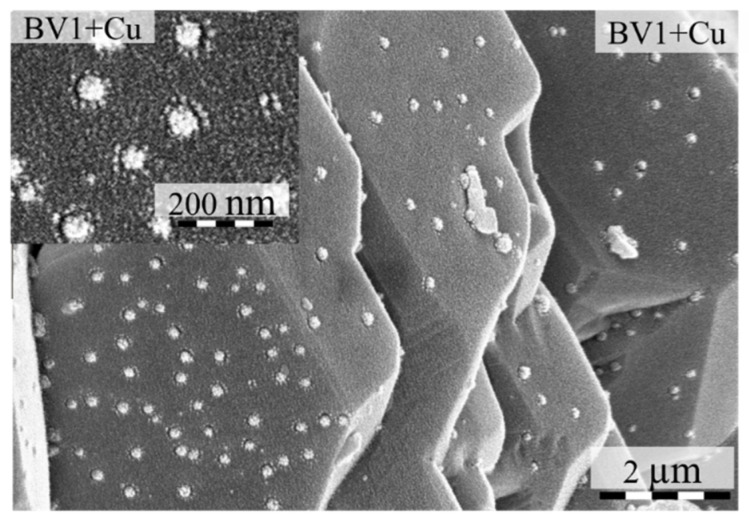
The Cu nanoparticles deposited on the surface of sample BV1.

**Figure A1:**
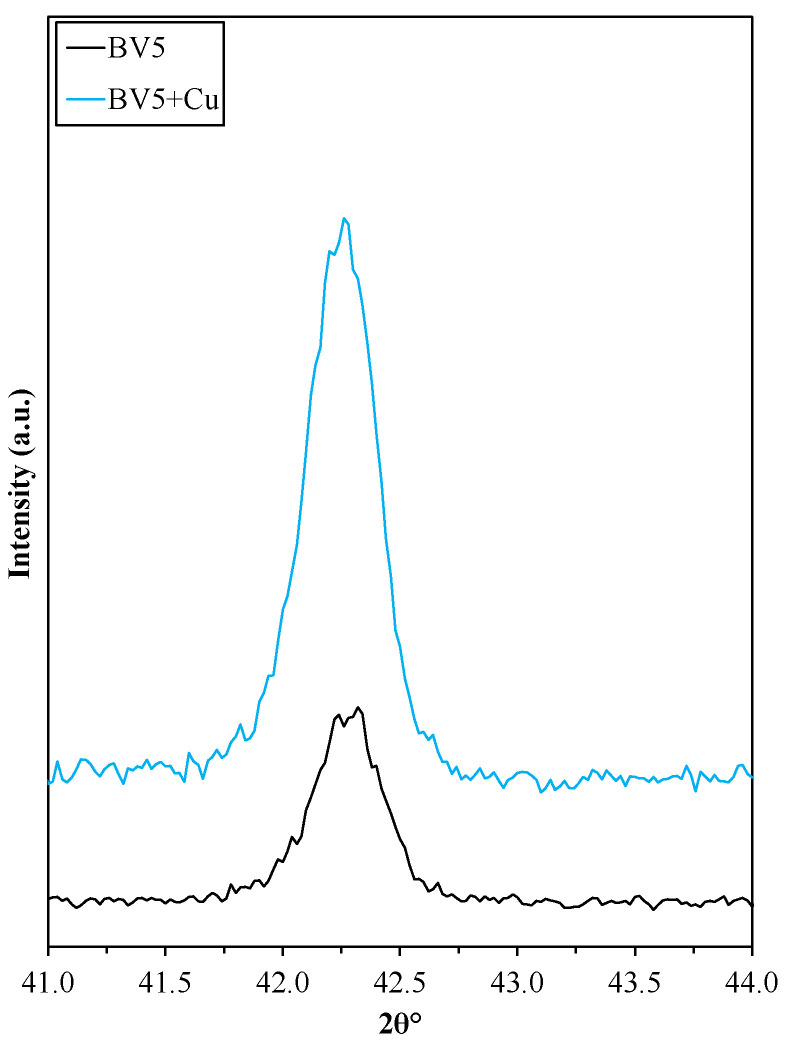
The diffraction pattern of sample with and without Cu.

The diffraction peaks of Cu, deposited on bismuth vanadate (Figure A1) samples, were the same as the ones without copper. The main diffraction peaks of Cu were covered by the signals of BiVO_4_, namely Cu(111) with BiVO_4_(051) and Cu(200) with BiVO_4_(202), therefore the presence of crystalline Cu nanoparticles was evident from the intensity differences registered at the mentioned BiVO_4_ diffraction peaks. The main morphology of Cu-BiVO_4_ samples did not change, but small Cu grains appeared at the surface of BiVO_4_. These small grains were also visible in the SEM micrographs as well, as shown in Figure 6.

**Figure 7:**
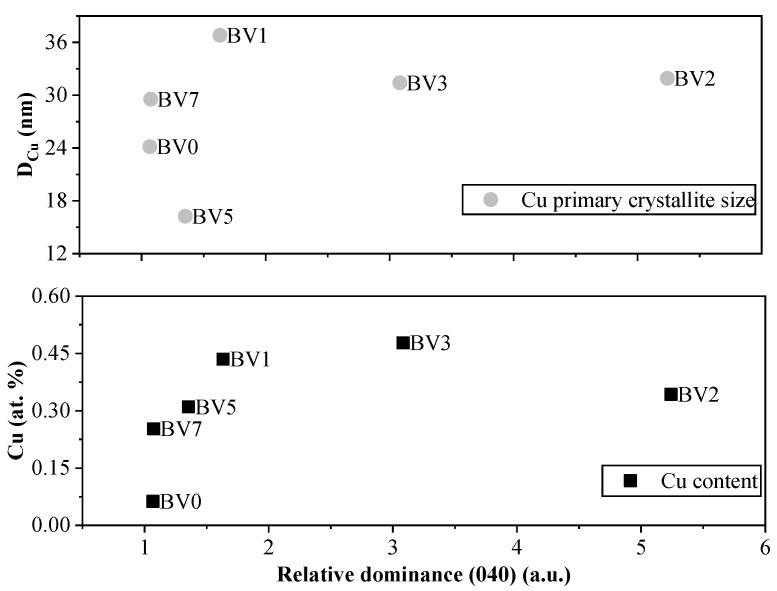
The dependence of the Cu deposition process on the surface of BiVO_4_. As the dominance of (040) increases so does the deposited amount of Cu and the Cu primary crystallite size as well.

The primary average crystallite size of these Cu grains continuously decreased, from 36.8 to 16.3 nm, as the pH value increased, except for samples BV0 and BV7 (Figure 7). The Cu amount in the Cu-BiVO_4_ samples follows the intensity of (040) crystal facet of BiVO_4_, except for samples BV9 + Cu and BV3 + Cu (Figure 7). However, sample BV9 cannot be considered here, as its base material is mostly Bi_2_O_3_.

It was interesting to see that the deposition of Cu nanoparticles (in terms of quantity and crystallite mean size) followed the trend obtained in the case of oxalic acid degradation, meaning that a selective reaction was occurring on the (040) crystallographic plane. However, this needed further confirmation, thus Raman spectroscopy measurements were carried out.

**Figure 8:**
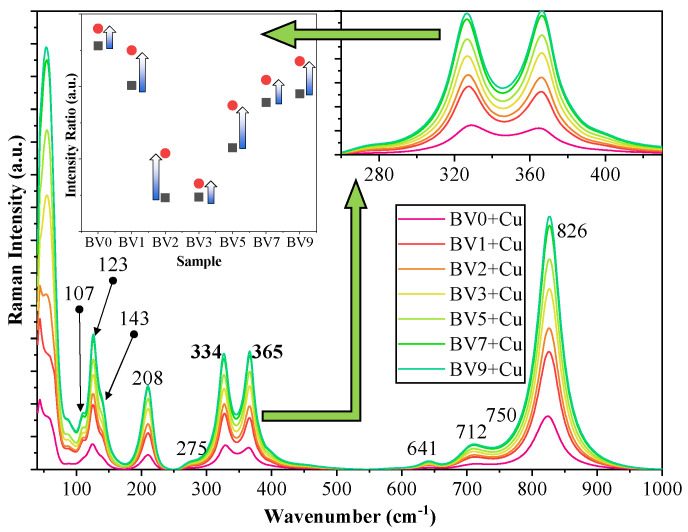
The Raman spectra of the Cu deposited BiVO_4_ photocatalysts. The inset shows the changes of the bending mode assigned peaks, with the catalyst type.

In the Raman spectra of the samples, several characteristic bands were assigned (Figure 8). At lower Raman shift values, namely below 100 cm^−1^ and around 123, 143, 208 and 275 cm^−1^, the specific lattice modes were identified, specific for monoclinic clinobisvanite. This was followed by the VO_4_^2−^ species υ_2_ bending vibrations (328 and 368 cm^−1^). The Bi-O stretching vibration was located at 641 cm^−1^, while υ_3_ antisymmetric stretching vibrations of VO_4_^2−^ species were identified at 712 and 750 cm^−1^, respectively. The broad and intense band located at 826 cm^−1^ can be associated with the υ_1_ symmetric stretching mode vibrations. In the case of VO_4_^2−^ species, υ_2_ vibrations were considered and analyzed. If structural changes were occurring in the samples which may affect the relative intensity of these vibrations, then they would have to show the same ratio change trend.

To deduce what kind of structural changes may occur in the samples, the following approaches can be considered:

- The main reaction is the reduction of Cu^2+^ to Cu.
- The two electrons used in the reduction process must originate from an oxidation reaction. In Bi-based photocatalysts, it is a known issue that Bi^3+^ can be oxidized to Bi^5+^ if a suitable reaction partner is present, which can be the above-mentioned reduction reaction.

If this reaction couple indeed occurs, then a specific change should be visible in the Raman spectrum of the samples, namely a unique shift in the ratio of the two bands should be visible while the general ratio between them is preserved. Indeed, the sought changes are visible in Figure 8. Both sample series (with or without Cu) followed the activity trend of the oxalic acid degradation (as function of the sample synthesis pH), while the two band ratio values of the Cu containing samples were generally higher. However, no correlations have been found thus far between rhodamine B degradation performances and a specific property of the BiVO_4_ materials.

### 2.6. DLS Measurements

**Figure 9:**
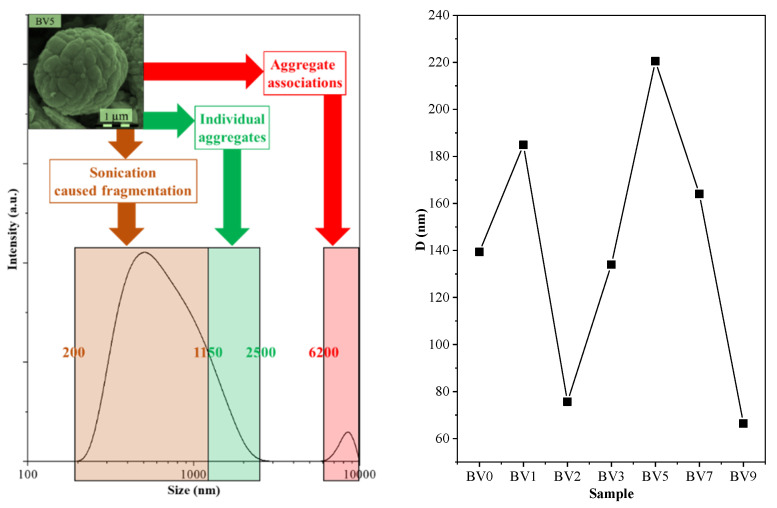
The size distribution of sample BV5, measured by DLS, showing a bimodal size distribution. The sample (synthesis pH) dependence of the hydrodynamic particle size.

Some semiconductor nanocrystals behave differently in aqueous medium. Photocatalytic materials are no exception. Furthermore, prior to photocatalytic measurements, usually a homogenization procedure is applied (e.g., ultrasonication), which may have an impact on the properties of the semiconductors. First, the hydrodynamic particle size was analyzed (Figure 9), and interestingly low values (60–220 nm) were obtained compared to the crystal size values determined by SEM (up to 5 µm). However, in the case of samples BV2 and BV3, additional peaks were observed, around 5.5 µm, which coincided with the double platelet size, and lower values (40–100 nm) were detected, which is nearly identical with the double plate thickness of these samples, meaning a pair association of these crystals. In other cases, such as the one mentioned above (Figure 9), three different zones were observed: one responsible for particle fragmentation, one showing the real size and one showing the associated microcrystals.

This means that the semiconductor particles did not endured the ultrasonication (2 h) procedure that was applied prior to the DLS measurements. It should be emphasized again that, without prior long time ultrasonication, it was not possible to measure real values for rapidly sedimenting samples. However, disintegrating larger crystals into smaller ones should not change their general behavior in water. Hence, the obtained DLS particle size was plotted against the sample synthesis pH/sample name. The obtained trend was nearly identical with the one obtained for rhodamine B. This means that, as the hydrodynamic particle size increased (higher agglomeration tendency) so did the visible light photoactivity. Although this result may be in contradiction with general trends (higher dispersity—larger available surface for the photocatalytic reactions—stable suspensions), it can be explained considering other approaches.

As the photodegradation of rhodamine B cannot be correlated with the presence of (040) crystallographic plane, it can be assumed that it is an important activity factor but not the only major one, as in the case of oxalic acid. As mentioned above, rhodamine B degradation includes an electron transfer step. A general electron transfer step in semiconductor photocatalysts is usually facilitated in hierarchical microcrystalline systems (e.g., in the case of TiO_2_ [45]), which assumes an interparticle charge transfer. In the present case, the particle aggregation state is the highest in those samples which showed the highest activity for rhodamine B: BV1 and BV5.

## 3. Materials and Methods

### 3.1. Materials

All chemicals were analytical reagents and used without further purification. Bismuth nitrate pentahydrate ((Bi(NO_3_)_3_∙5H_2_O, ≥98%) and sodium metavanadate (NaVO_3_, ≥98%) from Sigma-Aldrich (St. Louis, MO, USA), nitric-acid (HNO_3_, 69%) from Merck, oxalic acid (H_2_C_2_O_4_·2H_2_O, ≥99.9) from Scharlau, rhodamine B (C_28_H_32_ClN_2_O_3_) from ReAnal (purity ≥99.9%), cooper chloride dihydrate (CuCl_2_·2H_2_O, 99%) from Alfa Aesar (Karlsruhe, Germany)and high purity deionized water were used throughout the whole work.

### 3.2. Preparation of Differently Shaped BiVO_4_ Particles

In all cases, the molar ratio of Bi:V precursors was fixed to 1:1. The main procedure can be summarized as follows. Firstly, 2.5 mmol bismuth nitrate pentahydrate (1.212 g) was dissolved in 55.7 mL, 2 M HNO_3_ (solution A), while 2.5 mmol sodium metavanadate was dissolved in 55.7 mL deionized water (solution B). The two solutions were stirred continuously for 20 min at room temperature, and then solution B was added dropwise into solution A, under vigorous stirring. After the appearance of a yellow precipitate, the mixture was stirred for an additional 30 min. Thereafter, the pH value was adjusted with 10, 2 or 0.2 M NaOH solution, depending on the desired final pH values, which were 0.8, 1, 2, 3, 5, 7 and 9, respectively (please note that these values refer to the synthesis conditions and not the photodegradation experiment parameters).

After the pH setting procedure, the yellow suspension was sealed in a 172 mL Teflon-lined autoclave and heated at 180 °C for 15 h. Afterwards, it was allowed to cool down to room temperature. The obtained yellow powder was washed with absolute ethanol and deionized 5 times and finally dried at 80 °C for 24 h. The samples were coded as follows: BV0, BV1, BV2, BV3, BV5, BV7 and BV9, where the numbers represent the adjusted pH values. It is important to note that sample BV0 was obtained without adding NaOH (the pH value was ≈0.8)

### 3.3. The Selective Deposition of Cu^2+^ on BiVO_4_

To verify the crystallographic plane selectivity of non-photocatalytic reaction, Cu deposition was carried out on the surface of the BiVO_4_ photocatalysts [46]. Cu^2+^ was anchored on each bismuth vanadate sample by an impregnation method. More precisely, 10 mL of 0.1 M CuCl_2_·2H_2_O was added to 1 g of the as prepared BiVO_4_. Each suspension was heated to 90 °C for 1 h under intensive stirring. Afterwards, the solution was centrifuged, and the obtained powder was washed with distilled water. The obtained powder was dried at 110 °C for 24 h. The Cu containing samples were coded as follows: BV0 + Cu, BV1 + Cu, etc. The long-term stability, photocatalytic activity and attachment mechanism were not investigated, as they were not the subject of the present study.

### 3.4. Methods and Instrumentation

X-ray diffraction (XRD) measurements were applied to identify the crystalline phases and mean primary crystal size values of the samples. The XRD patterns were recorded on a Rigaku MiniFlex II diffractometer (Tokyo, Japan) using Cu-K_α_ radiation (λ = 0.15406 nm, 30 kV, 15 mA), equipped with a graphite monochromator. The diffraction data were recorded from 20° to 80° (2θ°). The scanning speed was 3 (2θ°) min^−1^. The average size of the crystals was calculated using the Scherrer equation [47], and the (121) diffraction peak was used for the calculation procedure. The main diffraction peaks of Cu were covered by the signals of BiVO_4_, namely Cu(111) with BiVO_4_(051) and Cu(200) with BiVO_4_(202), hence the primary crystallite size values were calculated using differentiation following a normalization process.

The particle size and distribution, as well as the morphology of the particles, were analyzed by cold field-emission scanning electron microscope (SEM), Hitachi S-4700 Type II (Tokyo, Japan). The applied accelerating voltage was 10 kV. Samples for SEM measurements were attached to a carbon adhesive tape, which was fixed to an aluminum sample holder.

JASCO-V650 spectrophotometer (Jasco, Tokyo, Japan) with an integration sphere (ILV-724) was used for measuring the DRS spectra of the samples (λ = 250–800 nm). To obtain the band-gap energy the reflectance data were converted to F(R) values according to the Kubelka-Munk theory [48]. The band gap was obtained from the plot of (F(R)∙E)^1/2^ in function of the exciting light. The diffuse reflectance spectra were transformed by first order derivative of wavelength (dR/dλ) [44]. The derivation was applied between 425 and 600 nm. By this procedure, the possible electron transition bands were evaluated as well.

To identify the structural changes induced by the Cu deposition on bismuth vanadate, Raman spectra were acquired by Thermo Scientific DXR Raman microscope, equipped with a diode-pumped frequency-doubled Nd:YAG laser with 10 mW maximum laser power (780 nm, spot size of approximately 1 µm). The acquired spectra were recorded at 2 cm^−1^, while a 50-µm slit confocal aperture was used for each measurement.

The particle size distribution and zeta-potential of the samples were measured using a Nano ZS90 Zetasiser analyzer (Malvern Instruments, Malvern, UK) equipped with a He-Ne laser (633 nm, 5 mW). Analyses were performed at a scattering angle of 90° and temperature of 25 °C. The sample was measured three times and the mean value is reported. The samples were sonicated for 2 h prior the measurements to assure a homogenous suspension during the measurements. The prolonged sonication was necessary to prevent the sedimentation of those particles which were not stable in the used suspensions.

The trace element content (in our case Cu) of the samples was measured with a Horiba Jobin Yvon XGT-5000 X-ray fluorescent spectrometer (Paris, France), equipped with Rh X-ray source. The records were made at 30 kV excitation voltage, 0.5 mA anode current and 1000 s measuring time.

### 3.5. Photocatalytic Activity

The photocatalytic activity was determined by the photodegradation of rhodamine B (RhB) and oxalic acid at 25 °C. Four 24-W conventional energy saving lamps with cutoff filter (λ > 400 nm) were used as a light source. The photocatalytic degradation experiments were performed as follows: 100 mg BiVO_4_ powder was added to 100 mL RhB solution (1 × 10^−5^ mol dm^−3^) or 100 mL oxalic acid solution (5 × 10^−3^ mol dm^−3^), and the catalysts were dispersed in the model pollutant solution using an ultrasonication bath for 5 min (the pH of the suspension was not adjusted). Before the start of the photocatalytic tests, the suspension was stirred in the dark for 30 min to achieve the adsorption/desorption equilibrium. After the lamps were switched on, a 2-mL suspension was collected and centrifuged every 30 min. The oxalic acid degradation was followed by an Agilent 1100 type high performance liquid chromatography (Santa Clara, CA, USA), which was equipped with an UV-Vis detector and a GromResin ZH type column (the eluent was 19.3 mmol L^−1^ H_2_SO_4_ with 0.8 mL∙min^−1^ flow rate, the detection wavelength was 206 nm). The rhodamine B concentration was determined by an Agilent 8453 UV-Vis spectrophotometer (detection wavelength = 553 nm). Oxalic acid was chosen as it is known to usually act as a hole scavenger in photocatalytic reactions, while Rhodamine B can be degraded via holes and both hydroxyl radicals as well.

## 4. Conclusions

In the case of photocatalytic materials, a one-way interpretation is carried out in most of the available literature, showing that a specific crystal morphology is responsible for the photoactivity. In the present study, the photodegradation of oxalic acid and rhodamine B (two model pollutants which show different photodegradation pathways) was carried out under visible light irradiation, and it was found that the photodegradation of oxalic acid was dependent from the presence of (040) crystallographic plane. The charge carrier exchange of (040) was proved by the Cu deposition reaction, reinforced by Raman spectroscopy. Furthermore, it was found that the aggregation state in aqueous media was the key in explaining the photoactivity towards rhodamine B.

In terms of morphology, it was found that the increase of the synthesis pH influences drastically influenced the thickness of the truncated octahedral particles, making the (040) crystallographic plane the dominant exposed one. As the pH value reached 5 the particles became polycrystalline and aggregates were formed (at this point, the affinity towards the pollutants also changed drastically), which at higher pH value are disintegrated. At pH 9, the upper limit of the series was reached as Bi_2_O_3_ started to precipitate instead of the desired BiVO_4_ causing an activity drop in all cases.

## Figures and Tables

**Table 1:** The structural, optical and photocatalytic properties of the BiVO_4_ crystals.

Sample Name	Crystal Size (nm)	Band Gap (ev)	dR/dλ_max_ (nm)	Degraded RhB (%)	Degraded Oxalic Acid (%)
BV0	42.8	2.10	527	-	-
BV1	37.4	2.31	517	21.21	38.16
BV2	36.3	2.30	501	43.20	51.13
BV3	36.9	2.35	497	23.27	47.86
BV5	29.8	2.37	498	61.34	37.58
BV7	33.0	2.40	506	51.11	38.62
BV9	43.4	2.20	519	21.49	41.82
